# The influence of ontogenetic dietary fluctuations on zebrafish size and swimming performance

**DOI:** 10.3389/fphys.2012.00310

**Published:** 2012-07-31

**Authors:** Chris Marks, Steven M. Lombardo, Kristie L. Formanik, Francisco B.-G. Moore, Brian Bagatto

**Affiliations:** Department of Biology, The University of AkronAkron, OH, USA

**Keywords:** zebrafish, *Danio rerio*, ontogeny, swim, quantitative genetics

## Abstract

Phenotypic flexibility is critical in determining fitness. As conditions change during ontogeny, continued responsiveness is necessary to meet the demands of the environment. Studies have shown that subsequent ontogenetic periods of development can interact with one another and shape developmental outcomes. The role genetic variation within populations plays in shaping these outcomes remains unclear. Four full-sib families of zebrafish *Danio rerio* were raised under for dietary regimes: high food rations for 60 days (HH), low food rations for 60 days (LL), high food rations for 30 days followed by low food rations for 30 (HL), and low food rations for 30 days followed by high food rations for 30 (LH). While the low ration diet significantly reduced body length at 30 days, diet was no longer a significant factor at day 60. Only family level variation influenced body length. Furthermore, there was significant family level variation in the manner in which swimming performance responded to fluctuating dietary conditions. Some families increased swimming performance in response to dietary change, while others did not. These results suggest that plastic responsiveness to subsequent environmental changes can be trait specific and vary significantly within populations.

## Introduction

Phenotypic plasticity is a critical aspect of organismal development. As conditions change during ontogeny, continued responsiveness is necessary to meet the demands of the environment. The nature of developmentally plastic responses are dependent upon the developmental window studied (Burggren and Reyna, 2011). For example, we previously demonstrated that hypoxia imposed early during ontogeny can later influence traits such as aggression, swimming performance, and lactate production in zebrafish (Marks et al., 2005; Widmer et al., 2006). Specifically, we found that fish tested in normoxia displayed phenotypes that were altered by development in early hypoxia.

Numerous studies have demonstrated that plastic responses vary across genotypes (DeWitt and Scheiner, 2004). This variation provides the raw material for selection to optimize developmental plasticity (West-Eberhard, 2005). Unfortunately, much of our current understanding of the genetics of developmental plasticity comes from studies tracking genotypes across one instance of environmental change. As the environment can change multiple times during ontogeny, it becomes increasingly important to characterize the role genetic variation plays in more complex environments.

Food availability is a critical factor in shaping animal development. Food manipulation studies have demonstrated significant effects of dietary change on fish metabolism and swimming performance (Beamish et al., 1989; Alsop and Wood, 1997). Food type and availability vary and such changes are associated with seasons (Wu and Culver, 1992), presence of competitors (Osenberg et al., 1992), and microhabitat use (García-Berthou, 1999). Ontogenetic dietary shifts have been demonstrated to increase cognitive performance in the cichlid *Simochromis pleurospilus* (Kotrschal and Taborsky, 2010). Fish that were switched from high-ration to low-ration and low-ration to high-ration diets outperformed conspecifics maintained on steady high- or low-ration diets. While dietary change clearly influences developmental outcomes in fish, the role genetic variation plays in influencing these altered responses in more complex environments remains unclear.

To elucidate the role genetic variation plays in determining responses to ontogenetic dietary fluctuations, we examined body size and swim performance in the zebrafish *Danio rerio*. Four full-sub families were fed either consistent high or low food rations, or a combination of the two for 60 days. We analyzed sources of variation on phenotypic outcomes as functions of family (F), early diet (days 0–30; Diet_0–30_), later diet (days 30–60; Diet_30–60_), interactions between dietary environments (Diet_0–30_ × Diet_30–60_), genetic variation to either dietary environment (F × Diet_0–30_, F × Diet_30–60_), and genetic variation in the response to interactions between dietary environments (F × Diet_0–30_ × Diet_30–60_). With many potential sources of variation, we made no a priori hypotheses on these sources.

## Materials and methods

### Animals

This experiment was performed under approval by The University of Akron's Institutional Animal Care and Use Committee. Adult zebrafish (*D. rerio*) were obtained from Aquatica Tropicals (Plant City, FL) and populations of wild-type strains were maintained according to standard procedures (Westerfield, 1994). Adults were maintained and bred at 26 ± 0.5°C with a 14L:10D light cycle.

### Breeding design

Males and females were randomly paired resulting in four full-sib families. Mating pairs were placed in 2-L containers lined with a marble substrate and supplied with a common water source (Z-Mod housing system, Marine Biotech, Beverly, MA).

### Treatments

Siblings were raised together in 2-L containers with a common water source (Z-Mod housing system, Marine Biotech, Beverly, MA) and were maintained at 26 ± 0.5°C with a 14L:10D light cycle for the duration of the experiment. Food consisted of pulverized Zeigler^TM^ adult zebrafish diet supplemented with equal parts of <100 and 100–150 micron Zeigler^TM^ larval diet (1:1:1). After 30 days, the <100 and 100–150 micron supplements were replaced with 150–250 and 250–450 micron supplements. For all feedings, 500 mg of food was mixed with 250 ml of system water. From this solution, fish were fed at 0.1 mg/fish (low-ration treatment) and 0.2 mg/fish (high-ration treatment). We chose these rations based on a standard dry food recipe from a protocol available at the Zebrafish International Resource Center (http://zebrafish.org/zirc/documents/protocols/pdf/Fish_Feeding/Flake_Food/Dry_Food_Recipes.pdf). We assigned 0.1 mg/fish to the low-ration treatment since it was the amount designated by this protocol. Therefore, the terms “high” and “low” are relative and apply only to the confines of this particular study. Feedings were conducted once daily and excess food was removed before each feeding. After two weeks, we noted all food was being consumed within 24 h. After 30 days, half of the individuals from each treatment were switched to a separate 2-L tank and subjected to the opposite feeding treatment for the remainder of the experiment. This resulted in four feeding treatments: high food rations for 60 days (HH), low food rations for 60 days (LL), high food rations for 30 days followed by low food rations for 30 (HL), and low food rations for 30 days followed by high food rations for 30 (LH). These four treatments were applied to all four families. Individuals from each family were housed together according to feeding treatments.

### Measurements

#### Body size

To ensure feeding treatments had an initial effect, we measured the total length (TL) of each subject at 30 days. Fish were placed individually in a small petri dish filled with system water. A ruler was included in each picture. Photography was conducted with a Nikon D300 camera under standard lighting conditions. We measured TL from the most anterior point to the posterior point of the caudal fin. We observed no damage to caudal fins at any point in the study. At 60 days, each subject was euthanized with MS-222 (300 mg/l tricaine methane sulfonate buffered to a neutral pH with sodium bicarbonate) and photographed on the subject's right side with a length standard in each picture. We measured standard length (SL) from the most anterior point to the base of the hypural plate at caudal flexion. Maximum depth (MD) was measured as the maximum dorsal–ventral distance measured along the flank. All measurements were made using ImageJ (Version 1.42, NIH). Measurements were made five times on each subject and the mean was recorded.

#### Swim velocity

Prior to terminal measurements (above), maximum swimming velocity (Umax) was measured according to Widmer et al. (2006). Briefly, individual fish were placed in a clear acrylic flume (44.7 mm inner diameter by 30 cm long) which drew system water. System water was maintained at 26.5°C with a Lifegard heater module (Pentair Aquatics). Water was oxygenated to 6.8 mg/l with airstone bubblers. Baffles placed at the anterior portion of the swim chamber maintained consistent laminar flow throughout the length of the flume. Individual subjects were allowed to acclimate to the tunnel for 5 min prior to measurement. With an initial flow velocity of 4 cm/s, flow (Blue-White Industries, Huntington Beach, CA, USA; flow rate meter F-1000-RB) was increased every 5 s by 2 cm/s until the fish spent >50% of the time increment touching the back mesh of the chamber (Brett, 1964). Maximum swim velocity was calculated based on the inner diameter of the tube and the final flow measurement and was determined based on the SL of the fish tested. Subjects were selected randomly from families/treatments.

### Statistics

To test for the effects of feeding for days 0–30 on TL, we used a Two-Way ANOVA. Family, treatment, and their interaction were included as sources of variation. To test for the effects of feeding throughout the experiment on SL and swim velocity, we used a Three-Way ANOVA. Family, food treatment for days 0–30 (Diet_0–30_), food treatment for days 30–60 (Diet_30–60_), and all possible interactions were included as potential sources of variation. For swimming velocity, comparisons across all families and treatments we made using Tukey's HSD. TL and SL were log transformed to meet normality assumptions. Statistics were performed in JMP version 9.0.2 (SAS institute).

## Results

### Survival

Ninety-three subjects survived the experiment. Chi-squared tests revealed that survival shared no contingencies with feeding treatments (*X*^2^ = 0.56, *P* = 0.91) or families (*X*^2^ = 2.91, *P* = 0.41). Sample sizes for treatments and families are shown in Tables 2 and 3.

### Body size

**Figure 1:**
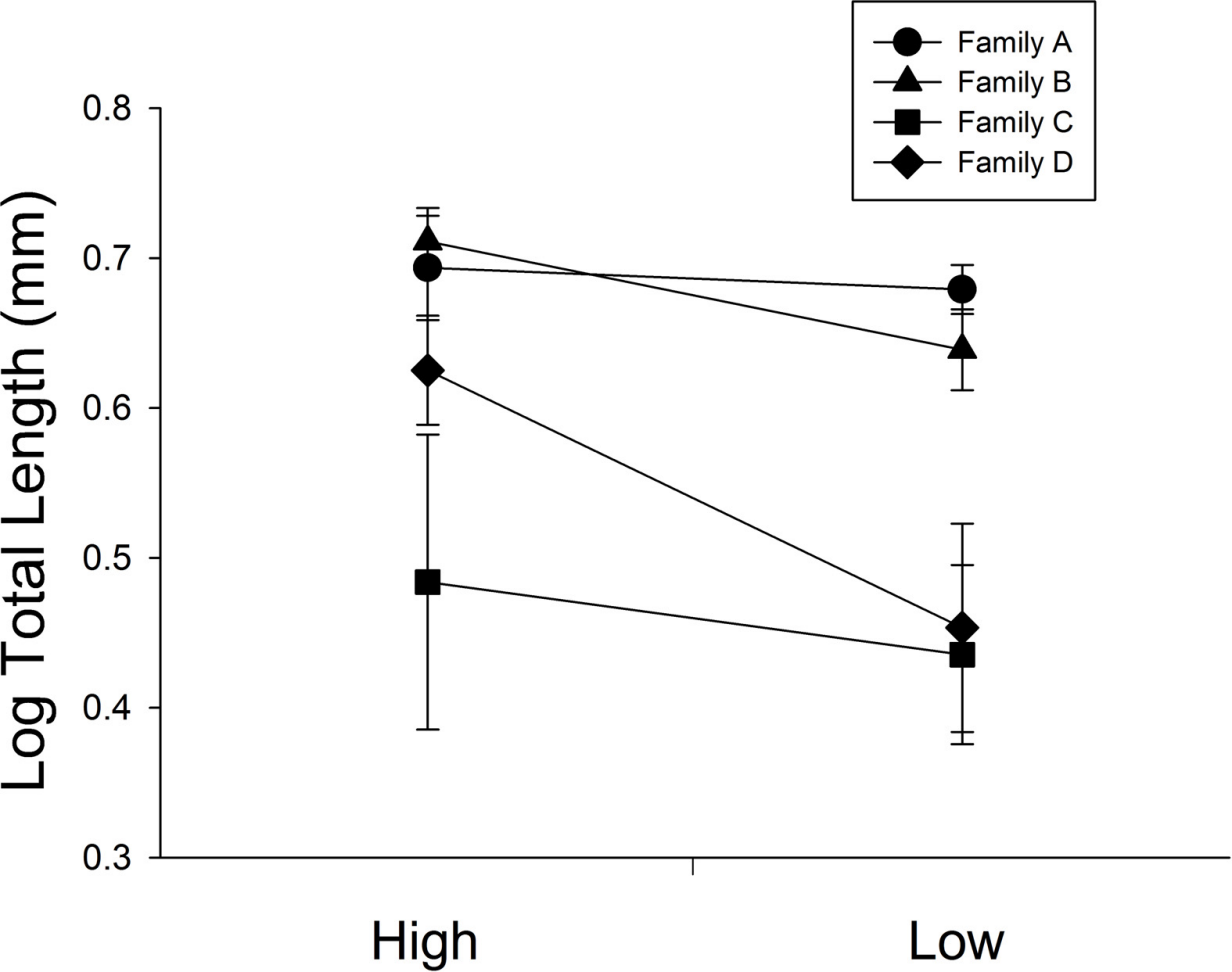
**The influence of diet ration (High or Low) for days 0–30 on total length in four full-sib families (A, B, C, and D; *n* = 34 per treatment)**. Error bars represent standard errors. Diet and family were significant sources of variation (*P* = 0.0026, <0.001, respectively).

At 30 days, family (F) and diet were both significant factors in influencing TL (Table 1). Individuals from the high food treatment were significantly longer (4.68 ± 0.20 mm vs 3.87 ± 0.17 mm; Figure 1). At 60 days, however, the only significant source of variation on SL and MD was family (Table 1). Mean standard lengths (untransformed) are presented in Table 2.

**Table 1:** **ANOVA results for total length (TL; *n* = 136), standard length (SL; *n* = 93), maximum depth (MD; *n* = 93), and swim velocity (*n* = 93)**.

**Variable**	**Source**	**DF**	**MS**	***F***	***P***
Total length	Family	3	16.4850	7.8045	<0.0001
	Diet_0–30_	1	19.8648	9.4046	0.0026
	Family × Diet_0–30_	3	0.7649	0.3621	0.7805
	Error	128	2.1122		
Standard length	Family	3	3.4353	245.04	<0.0001
	Diet_0–30_	1	0.0123	0.87	0.3526
	Family × Diet_0–30_	3	0.0017	0.12	0.9477
	Diet_30–60_	1	0.0424	3.02	0.0860
	Family × Diet_30–60_	3	0.0013	0.09	0.9645
	Diet_0–30_ × Diet_30–60_	1	0.0003	0.02	0.8861
	Family × Diet_0–30_ × Diet_30–60_	3	0.0078	0.56	0.6438
	Error	77	0.0140		
Maximum depth	Family	3	2.6945	242.74	<0.0001
	Diet_0–30_	1	0.0066	0.60	0.4426
	Family × Diet_0–30_	3	0.0055	0.50	0.6858
	Diet_30–60_	1	0.0370	3.33	0.0718
	Family × Diet_30–60_	3	0.0043	0.39	0.7612
	Diet_0–30_ × Diet_30–60_	1	0.0009	0.08	0.7724
	Family × Diet_0–30_ × Diet_30–60_	3	0.0074	0.67	0.5728
	Error	77	0.0111		
Swim velocity	Family	3	491.9777	54.31	<0.0001
	Diet_0–30_	1	7.1416	0.79	0.3774
	Family × Diet_0–30_	3	5.0020	0.55	0.6482
	Diet_30–60_	1	51.2372	5.66	0.0199
	Family × Diet_30–60_	3	28.1016	3.10	0.0315
	Diet_0–30_ × Diet_30–60_	1	56.8658	6.28	0.0143
	Family × Diet_0–30_ × Diet_30–60_	3	28.3586	3.13	0.0304
	Error	77	9.0594		

For TL, factors included family, diet for days 0–30 (Diet_0–30_), and their interaction. For SL, MD, and swim velocity, factors included family, diet for days 0–30 (Diet_0–30_), diet for days 30–60 (Diet_30–60_), and all possible interactions. TL, SL, and MD were log transformed for analyses.

**Table 2:** **Maximum depth and standard length (mm) for zebrafish (*n* = 93) at 60 days under all combinations of high and low food rations**.

	**HH (23)**	**HL (25)**	**LH (23)**	**LL (22)**
Maximum depth	5.86 ± 0.87	6.13 ± 0.90	5.84 ± 0.79	5.64 ± 0.82
Standard length	6.20 ± 0.96	5.32 ± 0.79	7.17 ± 0.97	6.10 ± 0.94

The first letter represents the food rations for days 0–30 (high vs low). The second letter represents the food rations for days 30–60 (high vs low). The number represents the sample size for each treatment. Data are presented as untransformed arithmetic mean ± SEM.

### Swimming velocity

**Figure 2:**
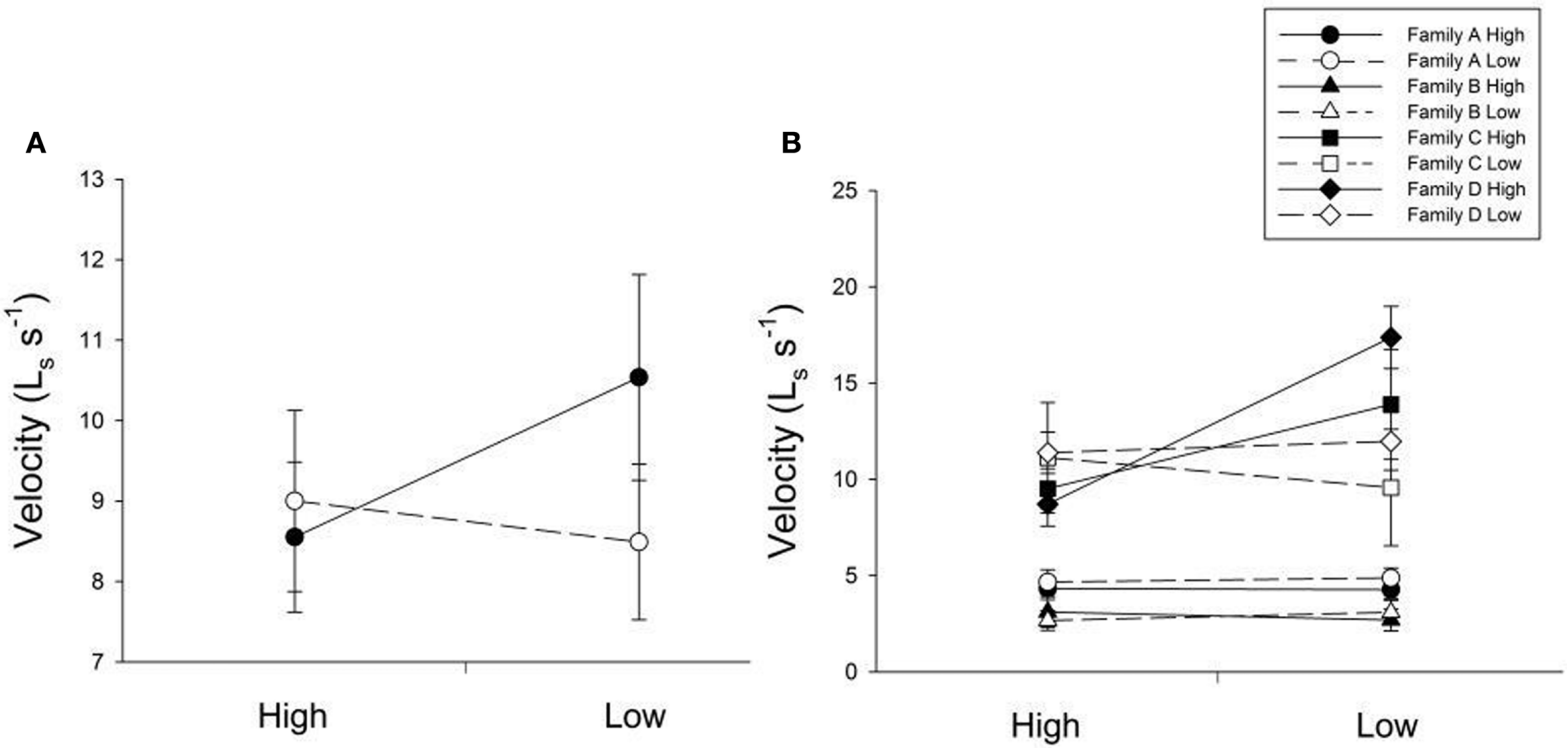
**(A)** The influence of changing diet ration (constant high, high-to-low, low-to-high, constant low) on swimming velocity across all four treatments. Open lines and symbols represent low food rations days 0–30 and solid lines and symbols represent high food rations for days 0–30. The x-axis represents food ration for days 30–60. Error bars represent standard errors. Diet for days 30–60 (Diet_30–60_) as well as an interaction between both dietary environments (Diet_0–30_ × Diet_30–60_) were significant sources of variation (*P* = 0.0199, 0.0143, respectively). **(B)** The influence of changing diet ration on swimming velocity in four full-sib families (A, B, C, and D; *n* = 93). Open lines and symbols represent low food rations days 0–30 and solid lines and symbols represent high food rations for days 0–30. The x-axis represents food ration for days 30–60. Error bars represent standard errors. Significant sources of variation included family (*P* < 0.001), the interaction between family and diet for days 30–60 (Family × Diet_30–60_; *P* = 0.0315), and the interaction between family and both dietary periods (Family × Diet_0–30_ × Diet_30–60_; *P* = 0.0304).

Many factors contributed to variation in swim velocity. Besides variation among families (F), diet for days 30–60 also contributed to variation in swim velocity (Diet_30–60_) and this effect varied significantly across families (F × Diet_30–60_; Table 1). Fish fed low rations for days 30–60 attained higher velocities on average than those fed high rations (Figure 2). Early diet (Diet_0–30_) also contributed to variation in swimming performance through its interaction with later diet (Diet_0–30_ × Diet_30–60_; Table 1). Fish raised on low rations for the duration of the experiment (LL) maintained similar velocities to those switched from low to high rations (LH; Figure 2A). Interestingly, fish switched from high to low rations (HL) attained higher swimming velocities than this maintained on high rations (HH; Figure 2A). The interaction between family and both dietary periods indicates that the quality of interactions (i.e., direction and magnitude) between dietary treatments varied across families (F × Diet_30–60_ × Diet_30–60_; Table 1). Variation in swim performance for families A and B remained consistent across diet treatments. Families C and D, however, showed variation due to both early (Diet_0–30_) and later (Diet_30–60_) food treatments. Fish from these families raised on low rations for the duration of the experiment (LL) maintained similar velocities to those switched from low to high rations (LH). Fish switched from high to low rations (HL), however, attained higher swimming velocities than those maintained on high rations (HH; Figure 2B). Comparisons across all families and treatments are show in Table 3.

**Table 3:** **Tukey HSD comparisons of swimming velocities across all families and treatments**.

	**A:HH**	**A:HL**	**A:LH**	**A:LL**	**B:HH**	**B:HL**	**B:LH**	**B:LL**	**C:HH**	**C:HL**	**C:LH**	**C:LL**	**D:HH**	**D:HL**	**D:LH**	**D:LL**
	**6**	**7**	**6**	**5**	**5**	**7**	**6**	**5**	**6**	**6**	**6**	**7**	**6**	**5**	**5**	**5**
A:HH		NS	NS	NS	NS	NS	NS	NS	NS	*	*	NS	NS	*	*	*
6															
A:HL			NS	NS	NS	NS	NS	NS	NS	*	*	NS	NS	*	*	*
7															
A:LH				NS	NS	NS	NS	NS	NS	*	NS	NS	NS	*	*	*
6															
A:LL					NS	NS	NS	NS	NS	*	NS	NS	NS	*	*	*
5															
B:HH						NS	NS	NS	*	*	*	NS	NS	*	*	*
5															
B:HL							NS	NS	*	*	*	NS	NS	*	*	*
7															
B:LH								NS	*	*	*	*	NS	*	*	*
6															
B:LL									*	*	*	NS	NS	*	*	*
5															
C:HH										NS	NS	NS	NS	*	NS	NS
C:HL											NS	NS	NS	NS	NS	NS
6															
C:LH												NS	NS	NS	NS	NS
6															
C:LL													NS	*	NS	NS
7															
D:HH														*	NS	NS
6															
D:HL															*	NS
5															
D:LH																NS

The first row and column represents each family and treatment. The first letter represents the family. The second letter represents the food rations for days 0–30 (high vs. low). The third letter represents the food rations for days 30–60 (high vs. low). The number represents the sample size for each family/treatment group. Significant comparisons are noted with an asterisks.

## Discussion

Low-diet fish were significantly smaller than high-diet fish at 30 days. This confirms that our feeding treatment significantly altered development prior to switching feeding treatments. Interestingly, neither nutritional environment contributed to variation in SL or MD at the end of the experiment. In other words, while early diet initially influenced size, subsequent nutritional change resulted in equal sizes across all four treatments. This indicates some compensatory growth mechanism for fish exposed to low food rations for days 0–30. Compensatory growth is generally not without cost. Following exposure to suboptimal conditions, compensatory growth has been shown to be associated with costs in a number of physiological, morphological, life history, and performance traits (reviewed in Metcalfe and Monaghan, 2001). Of particular interest is the finding that fish raised on constant low diet attained sizes similar to fish from the other feeding treatments. Although fish on low-ration diets were significantly smaller at 30 days, their growth rate had fully compensated by day 60. This suggests that resource allocation in this study was dependent upon the ontogenetic period, with resource allocation toward somatic growth increasing during days 30–60.

The performance trait addressed in this study was maximum swim velocity. Although they exhibited compensatory growth, fish raised on early low food rations maintained similar swimming velocity regardless of later dietary rations. This indicates negligible cost of compensatory growth on swimming performance in our study. Previous studies have demonstrated a tradeoff between accelerated growth and physical performance in salmon (Farrell et al., 1997) and sticklebacks (Álvarez and Metcalfe, 2007). In the case of sticklebacks, however, the associated tradeoff was present in stream rather than pond populations. This result indicates that local selective pressures can alter tradeoff trajectories among populations. Zebrafish inhabit a wide variety of habitats ranging from active streams to stagnant rice fields (Spence et al., 2008). It is therefore likely that ecological variation has shaped tradeoff trajectories in this species.

Interestingly, swim velocity was highest in fish switched from high to low food rations. Thus, dietary change enhanced swimming performance, but only for fish started on high rations. Fish started on low rations did not increase swimming velocity when switched to high rations. Thus, although HL and LH fish attained similar size, their physical abilities differed. Phillips (2004) performed a similar dietary switching study with mussels and found similar quantitative results. While mussels switched from high to low rations equaled those switched from low to high in terms of shell size, they differed in terms of lipid content. This result suggests that dietary order can be more critical in shaping physiological rather than morphological outcomes. Thus, the underlying physiologies of the subjects in our study may have been affected. Specifically, fish switched from high to low rations proved physiologically superior to fish from other treatments.

It should be noted that individuals from each family/treatment combination were housed together. Therefore, there remains the possibility of some influence of common rearing environment on each treatment group. This could be due to unique interactions between siblings of each family/treatment group. While we attribute the variation in this study to family and treatment effects, we do so with the understanding that these observations may be confounded. Although shared rearing space may be a component of variation in this study, we remain confident that family and treatment effects are major contributors to the observed variation. One reason for this is that mortality did not vary significantly among families or treatments. This suggests that the quality of interactions (aggressive encounters, food competition) were similar across families/treatments. However, we encourage further studies of the effects of changing dietary rations on fish physiology and physical performance to clarify these issues.

Swimming ability is a critical trait in fish. Its implications on prey capture, predator avoidance, and social interactions are evident (Videler, 1993). Thus, as swimming ability is sensitive to environmental change, the ontogenetic history of fish becomes critical in shaping their fitness. At the population level, variation in environmentally altered developmental trajectories provides the raw material for natural selection to optimize fitness in changing environments. Zebrafish inhabit a wide variety of habitats throughout Southeast Asia (Spence et al., 2008). Their association with a number of different habitats throughout seasonal fluctuations makes it likely that factors such as temperature, oxygen, and food availability can vary during their ontogeny. Thus, it is likely that there is some ecological component to variation in swimming ability in zebrafish. Given their small size, zebrafish are prolific swimmers that display remarkably low associated physiological costs (Plaut and Gordon, 1994). The selective factors that have shaped these abilities require further elucidation.

The role environmental complexity plays in shaping ontogenetic trajectories is receiving increasing attention. Of particular interest are the consequences multiple instances of environmental change have on developmental outcomes (Monaghan, 2008). Few studies to our knowledge have quantitatively demonstrated significant interactions between subsequent ontogenetic periods of development (Marks et al., 2005; Kotrschal and Taborsky, 2010). Even less clear is the role genetic variation plays in determining the quality of phenotypic outcomes under complex conditions. Our study not only demonstrates that subsequent dietary conditions can interact in shaping zebrafish physical performance, but the quality of these effects is family specific. This result indicates at least some role of genetic variation in shaping plastic responses under complex conditions. Such variation underlies the proximate variation necessary for selection to optimize developmental outcomes in changeable environments.

In summary, we found a significant interaction between dietary environments (Diet_0–30_ × Diet_30–60_) for swimming velocity. Overall, fish switched from high to low food rations attained the highest swimming velocity. Fish started on low food rations attained similar swimming velocities regardless of later food rations. The quality of responses to dietary change varied across families, resulting in a significant Family × Diet_0–30_ × Diet_30–60_ interaction. Although early food rations influenced size at the midway point of the experiment, fish achieved equal sizes across all food treatments at the end of the experiment. These results suggest that plastic responsiveness to subsequent environmental changes can be trait specific and vary significantly within populations. The specific order of environmental conditions can also be critical in determining performance outcomes.

### Conflict of interest statement

The authors declare that the research was conducted in the absence of any commercial or financial relationships that could be construed as a potential conflict of interest.

## References

[B1] Alsop D. Wood C. (1997). The interactive effects of feeding and exercise on oxygen consumption, swimming performance and protein usage in juvenile rainbow trout (*Oncorhynchus mykiss*). J. Exp. Biol. 200, 2337–2346 9320259 10.1242/jeb.200.17.2337

[B2] Álvarez D. Metcalfe N. B. (2007). The tradeoff between catch-up growth and escape speed: variation between habitats in the cost of compensation. Oikos 116, 1144–1151

[B3] Beamish F. W. H. Howlett J. C. Medland T. E. (1989). Impact of diet on metabolism and swimming performance in juvenile lake trout, *Salvelinus namaycush*. Can. J. Fish. Aquat. Sci. 46, 384–388

[B4] Brett J. R. (1964). The respiratory metabolism and swimming performance of young sockeye salmon. J. Fish Res. Board Can. 21, 1183–122

[B5] Burggren W. W. Reyna K. S. (2011). Developmental trajectories, critical windows and phenotypic alteration during cardio-respiratory development. Respir. Physiol. Neurobiol. 178, 13–21 10.1016/j.resp.2011.05.001 21596160

[B6] DeWitt T. J. Scheiner S. M. (eds.). (2004). Phenotypic Plasticity. Functional and Conceptual Approaches. New York, NY: Oxford University Press

[B7] Farrell A. P. Bennett W. Devlin R. H. (1997). Growth-enhanced transgenic salmon can be inferior swimmers. Can. J. Zool. 75, 335–337

[B8] García-Berthou E. (1999). Food of introduced mosquito fish: ontogenetic diet shift and prey selection. J. Fish Biol. 55, 135–147

[B9] Kotrschal A. Taborsky B. (2010). Environmental change enhances cognitive abilities in fish. PLoS Biol. 8:e1000351 10.1371/journal.pbio.1000351 20386729 PMC2850384

[B10] Marks C. West T. N. Bagatto B. Moore F. B.-G. (2005). Developmental environment alters conditional aggression in zebrafish. Copeia 2005, 901–908

[B11] Metcalfe N. B. Monaghan P. (2001). Compensation for a bad start: grow now, pay later? Trends Ecol. Evol. 16, 254–260 10.1016/S0169-5347(01)02124-3 11301155

[B12] Monaghan P. (2008). Early growth conditions, phenotypic development and environmental change. Philos. Trans. R. Soc. Lond. B Biol. Sci. 363, 1635–1645 10.1098/rstb.2007.0011 18048301 PMC2606729

[B13] Osenberg C. W. Mittelbach G. G. Wainwright P. C. (1992). Two-stage life histories in fish: the interaction between juvenile competition and adult performance. Ecology 73, 255–267

[B14] Phillips N. E. (2004). Variable timing of larval food has consequences for early juvenile performance in a marine mussel. Ecology 85, 2341–2346

[B15] Plaut I. Gordon M. (1994). Swimming metabolism of wild-type and cloned zebrafish *Brachydanio rerio*. J. Exp. Biol. 194, 209–223 9317659 10.1242/jeb.194.1.209

[B16] Spence R. Gerlach G. Lawrence C. Smith C. (2008). The behaviour and ecology of the zebrafish, *Danio rerio*. Biol. Rev. Camb. Philos. Soc. 83, 13–34 10.1111/j.1469-185X.2007.00030.x 18093234

[B17] Videler J. J. (1993). Fish Swimming. London, UK: Chapman and Hall

[B18] West-Eberhard M. J. (2005). Developmental plasticity and the origin of species differences. Proc. Natl. Acad. Sci. U.S.A. 102, 6543–6549 10.1073/pnas.0501844102 15851679 PMC1131862

[B18a] Westerfield M. (1994). The Zebrafish Book. Eugene, OR: University of Oregon Press

[B19] Widmer S. Moore F. B.-G. Bagatto B. (2006). The effects of chronic developmental hypoxia on swimming performance in zebrafish. J. Fish Biol. 69, 1885–1891

[B20] Wu L. Culver D. A. (1992). Ontogenetic diet shift in Lake Erie age-0 yellow perch (*Perca flavescens*): a size-related response to zooplankton density. Can. J. Fish. Aquat. Sci. 49, 1932–1937

